# Plant-based dietary patterns and fasting insulin: a cross-sectional study from NHANES 2017–2018

**DOI:** 10.1186/s40795-023-00780-3

**Published:** 2023-11-03

**Authors:** Dana Curlin, Marion E. Hare, Elizabeth A. Tolley, Justin Gatwood

**Affiliations:** 1 University of Tennessee Health Science Center, Memphis, TN USA; 2 College of Medicine – Department of Preventive Medicine, Division of Preventive Medicine, Memphis, TN USA; 3 College of Pharmacy – Department of Clinical Pharmacy & Translational Science, Nashville, TN USA

**Keywords:** Vegetarian diet, Vegan diet, Diet index, Fasting insulin, Body mass index, Alanine aminotransferase, National health and examination survey

## Abstract

**Background:**

Previous studies have created plant-based diet indices to assess the health effects of specific dietary patterns.

**Objective:**

To examine the association between the plant-based content of diet and fasting insulin in adults from the NHANES 2017–2018 database.

**Methods:**

Demographic, dietary, lab and clinical data and fasting insulin were obtained from the NHANES 2017–2018 database. From two 24-h dietary recalls, we created a plant-based diet index (PDI) and a healthy plant-based diet index (hPDI). A high PDI score indicated more plants were consumed versus animal foods. A high hPDI score indicated healthier, plant materials (whole grains, whole fruits, vegetables, legumes, vegetable oils, seeds and nuts) were consumed. The relationships between the natural log of fasting insulin, PDI, and hPDI were analyzed using multiple linear regression adjusting for body mass index (BMI) and alanine aminotransferase (ALT).

**Results:**

Analyses were based on 1,714 participants, 897 women and 817 men with a median age of 52 years. In this sample, 610 (35.6%) were white, 407 (23.8%) were black, 231 (13.5%) were Mexican, 207 (12.1%) were Asian, 157 (9.2%) were other Hispanic, and 102 (6%) were other or mixed race. Median fasting insulin was 9.74 μU/mL (IQR: 6.2, 15.56). For every 1 unit increase in PDI, the natural log of fasting insulin decreased 0.0068 ± 0.003 μU/mL (CI: -0.00097, -0.013) (*p* = 0.02). After adjusting for BMI and ALT, the PDI did not significantly predict fasting insulin as the association was not robust due to multicollinearity. The hPDI was inversely and significantly associated with the natural log of fasting insulin (-0.0027 ± 0.00134, CI: -0.000087, -0.0053) (*p* = 0.043) in a multivariable model including BMI and ALT.

**Conclusion:**

A healthy plant-based diet is associated with a decrease in fasting insulin levels. Healthfulness of the diet is an important factor when considering the benefit of a plant-based diet.

## Introduction

Diet consists of combinations of different foods rather than single ingredients or nutrients. A dietary pattern based on whole vegetables, whole fruits, nuts, seeds, legumes, and whole grains has been associated with numerous health benefits and has been recommended for chronic disease prevention [1, 2]. Plant-based diets have been found to decrease risk of all-cause mortality [3–6], coronary heart disease [4, 6–9], type 2 diabetes [9–14], cancer [15, 16], cardiometabolic disease [17, 18], weight gain [13, 19], and chronic kidney disease [20]. The USDA 2015–2020 Dietary Guidelines for Americans recommends healthy vegetarian diets as one of three healthful dietary patterns appropriate for all life stages [1].

Insulin resistance is a pathological condition, often a consequence of excess body weight, in which cells fail to respond to insulin, resulting in high glucose in the tissues. Progression of insulin resistance often leads to metabolic syndrome, nonalcoholic fatty liver disease, and type 2 diabetes. Lifestyle modification, including dietary change, is often recommended to combat insulin resistance. An increasing body of evidence shows that vegetarian diets can reduce insulin resistance [8, 21–23]. Hyperinsulinemia and insulin resistance are associated with an increased risk of type 2 diabetes, obesity, cardiovascular disease, cancer, and premature mortality [24]. In the face of hyperinsulinemia, insulin resistance occurs as tissues take protective measures against the damaging effects of hyperglycemia. Recent study of this cascade implicates hyperinsulinemia as a cause, not a consequence, of insulin resistance [24, 25]. Per the Adventist Health Study 2, a vegetarian dietary pattern reduced the risk of developing metabolic syndrome by 56% and reduced metabolic risk factors, including elevated systolic and diastolic blood pressures, triglycerides, glucose, and waist circumference, compared to a nonvegetarian diet pattern [26].

Dietary pattern analysis seeks to capture the combined effects of eating behaviors. To assess total mortality reduction from eating more plant-based foods, Martinez-Gonzalez et al. (PREDIMED) created a provegetarian index to rank the diets by consumption of plant-based foods of 7,216 Spanish adults with high cardiovascular risk using a repeated measures semi-quantitative food-frequency questionnaire. Fruit, vegetables, nuts, cereals, legumes, olive oil, and potatoes were weighed positively, while animal fats, eggs, fish, dairy products, meat, and meat products were weighed negatively. Participants with higher provegetarian index scores experienced lower mortality than those with low provegetarian scores over a median 4.8-year follow-up [3].

Recognizing that not all plant-based foods are healthy foods, Satija, et al. expanded upon the provegetarian indices by creating healthy versus unhealthy plant-based indices using repeated measures semi-quantitative food frequency questionnaires from the Nurses’ Health Studies and Health Professionals Follow-up Study. Analyzing a plant-based diet index (PDI), a healthy plant-based diet index (hPDI), and an unhealthy plant-based diet index (uPDI), Satija et al. found that the healthy plant-based diet (hPDI), emphasizing whole grains, fruits, vegetables, nuts, legumes, vegetable oils, and tea and coffee, had the strongest inverse associations with coronary heart disease and type 2 diabetes risks. Conversely, the uPDI, including juices, sweetened beverages, refined grains, potatoes or fries, sweets, and animal foods, was positively associated with coronary heart disease and type 2 diabetes risk [4, 7, 10]. Kim, et al. used NHANES III data, which utilized the food frequency questionnaire to gather dietary data, and reported an inverse association between a healthy plant-based diet and reduced all-cause mortality [5, 6]. These studies suggest that the quality of the plant foods is essential in conferring health benefits.

We used NHANES repeated measures dietary recall data to create a plant-based diet index (PDI) score to examine the associations between plant-based diets, specifically healthy and unhealthy plant-based diets, and fasting insulin. We hypothesized that individuals closely adhering to a plant-based diet would present with reduced fasting insulin, a biomarker indicative of metabolic dysfunction. Creating indices similar to those used in previous studies, we sought to analyze the benefit of adding healthful plant-based foods (foods low or without refined grains and sugar, emphasizing brightly colored vegetables, whole fruits, legumes, nuts and seeds, and vegetable oils) by creating a healthful, plant-based diet index (hPDI) in addition to a plant-based diet index (PDI). While most dietary studies examining dietary patterns have utilized semi-quantitative food frequency questionnaires, this study is one of few to utilize 24-h dietary recalls.

## Methods

The National Health and Nutrition Examination Survey (NHANES) is an on-going survey of the health and nutritional status of children and adults in the United States, conducted by the National Center for Health Statistics (NCHS), a part of the Centers for Disease Control (CDC). This survey combines interviews and physical examinations to assess health and nutritional status and generate health statistics for the Nation [27]. NHANES data has been approved by the NCHS Ethics Review Board (ERB), and informed consent was obtained in writing from all participants in this national survey [28]. Questionnaire instruments are published on the CDC website [29].

We examined de-identified data provided by the Centers for Disease Control and Prevention (CDC) National Health and Nutrition Examination Survey (NHANES) 2017–2018 to calculate a plant-based diet score, from a sample population in the United States, using the Food Patterns Equivalents Database (FPED) provided by the United States Department of Agriculture (USDA) Agricultural Research Service. Laboratory and demographics data were matched by respondent participant number to the two 24-h dietary food recall data sets included in the USDA FPED database What We Eat in America (WWEIA) (https://www.ars.usda.gov/ARSUserFiles/80400530/pdf/fped/FPED_1718.pdf; https://www.cdc.gov/nchs/nhanes/wweia.htm).

Beginning in 2002, the Continuing Survey of Food Intakes by Individuals and the NHANES dietary component merged to form consolidated dietary data named What We Eat in America (WWEIA), consisting of 2 days of 24-h recall data collected using the USDA’s Automated Multiple-Pass Method. The USDA and the National Center for Health Statistics jointly provide WWEIA. The Food and Nutrient Database for Dietary Studies (FNDDS) 2017–2018 provides nutrient composition for about 7100 foods and beverages in WWEIA, NHANES 2017–2018. The FPED includes the amounts of fruits, vegetables, grains, protein foods, dairy, oils, added sugars, solid fats, and alcoholic drinks present in 100 g of each of the FNDDS foods [1]. NHANES collects data in household interviews, at a mobile examination center (MEC), and in post-MEC follow up visits throughout the year including both weekdays and weekends [30]. The MEC was open 5 days per week and non-operational days were changed on a rotating basis, such that data encompassed all days of the week. Two examination sessions were conducted daily with participants randomly assigned to either morning or evening session. Dietary food recall capturing 24-h was collected in the NHANES MEC (day 1) and by telephone (day 2) 3 to 10 days later. The 24-h recall method is most often used for determining dietary intake in large-scale surveys [30, 31].

During each interview, measuring guides, useful in portion size estimation, were provided to assist in quantifying consumption. When day-to-day variation was considered, 24-h recalls were found to be comparable to FFQs with respect to estimates for intakes [32]. Use of the USDA’s Automated Multiple-Pass Method (AMPM) and collection of a second day of dietary recall accounts for day-to-day variation [30].

### Study population

 We included data from adult patients who had completed two days of 24-h recall and had fasted a minimum of eight hours prior to a fasting insulin measurement being drawn on day 1. Patients who were pregnant or had received insulin were omitted from analysis. Fasting insulin was measured among participants aged 12 and older who were examined in the morning session.

**Fig. 1:**
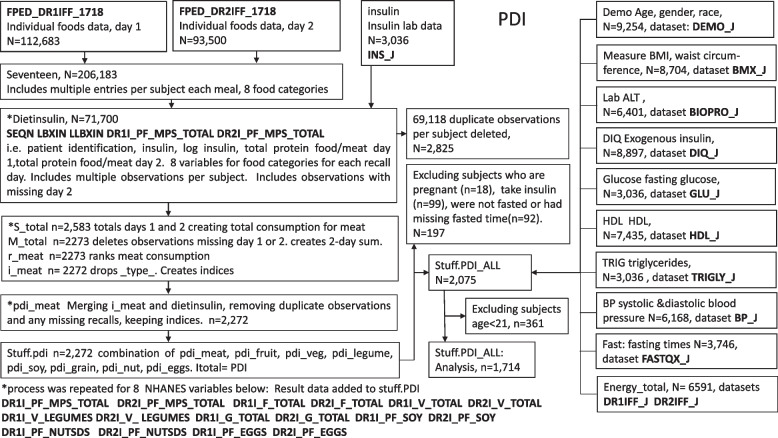
Flow diagram of selection of study participants and variables for PDI (NHANES filenames shown in bold)

**Fig. 2:**
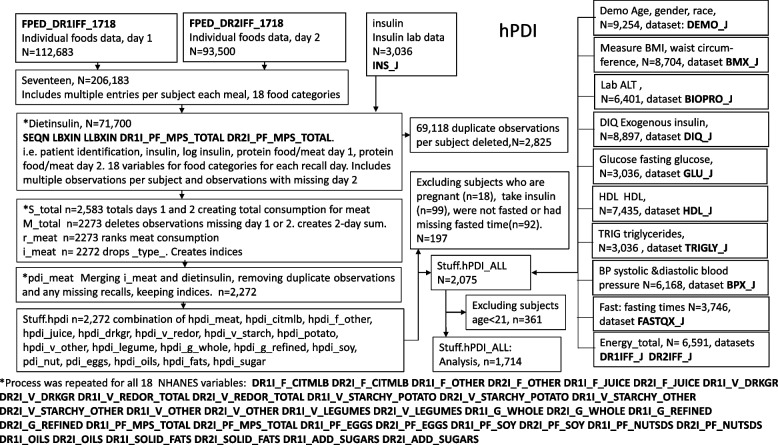
Flow diagram of selection of study participants and variables for hPDI (NHANES filenames shown in bold)

This cross-sectional study included 1,714 men and women aged 21 to 80 who all had fasting insulin and dietary data, BMI measurements were recorded in 1,537 of these subjects. Flow diagrams provided in Figs. 1 and 2 represent the selection of participants and variables. Survey weights were not applied since this study only examined one survey interval.

### Plant-based diet indices

Flow diagrams provided in Figs. 1 and 2 represent the selection variables included in the PDI and hPDI. Tables 1 and 2 (pages 31 and 32) list the variables from FPED included in the PDI (8 categories) and hPDI (18 categories), respectively.

**Table 1:** Variables from FPED constituting the PDI (8 categories)

*plant versus animal*		
***Category***	***FPED variable***	***Score: PDI***
Total fruit	F_TOTAL	positive
Total vegetable	V_TOTAL	positive
Legumes	V_LEGUMES	positive
Total grains	G_TOTAL	positive
Total meat poultry seafood	PF _MPS_TOTAL	negative
Eggs	PF_EGGS	negative
Soy	PF_SOY	positive
Nuts and seeds	PF_NUTSDS	positive

**Table 2:** Variables from FPED constituting the hPDI (18 categories)

*Healthy vs. unhealthy, plant/animal*		***Score:***
***Category***	***FPED variable***	***hPDI***
Citrus, melons, and berries	F-CITMLB	positive
Other fruits	F_OTHER	positive
Fruit juice	F_JUICE	negative
Dark green vegetables	V_DRKGR	positive
Total red& orange vegetables	V_REDOR_TOTAL	positive
White potatoes	V_STARCHY_POTATO	negative
Other starchy vegetables	V_STARCHY_OTHER	positive
Other vegetables	V_OTHER	positive
Beans and peas	V_LEGUMES	positive
Whole grains	G_WHOLE	positive
Refined grains	G_REFINED	negative
Total meat	PF_MPS_TOTAL	negative
Eggs	PF_EGGS	negative
Soy products-excludes soy milk	PF_SOY	positive
Nuts and seeds	PF_NUTSDS	positive
Oils-mostly plant based	OILS	positive
Solid fats- mostly animal based	SOLID FATS	negative
Added sugars	ADD_SUGARS	negative

Using the FPED 2017–2018 data, we calculated an overall plant-based diet index score (PDI) and a healthy plant-based diet index score (hPDI) using methods similar to previous studies by Martinez-Gonzalez, et al. and Satija, et al. [3, 10]. Food groups were ranked into quintiles by consumption and then scored positively (1 to 5) for plant-based and in reverse for animal-based (-1 to (-5)). Food groups with highest plant consumption received a 5; those with the lowest or no plant consumption received a 0. Likewise, food groups with the highest animal-food consumption received a (-5); those with the least animal consumption received a 0. PDI included 8 categories where plants were scored positively, and animal was scored negatively. Dairy was omitted as it included both non-mammal (soy, flax, etc.) and animal milk and could not be segregated. Sugar and fats were also omitted from the PDI indices but were included in the hPDI. Fruit juice was included in the total fruit category in the PDI; in the hPDI fruit juice consumption was separated from whole fruit. Alcohol, tea, and coffee were omitted from all indices.

The hPDI consisted of 18 categories designated as healthy (plant) and unhealthy (animal or processed plant) products. White potatoes, refined grain, fruit juice, solid fats, and added sugars were reverse scored as unhealthy. Vegetable oils were scored positively as healthy plant-based foods, while solid fats were reverse scored as unhealthy animal-based foods. Coffee/tea was not included as a FPED component and was omitted from both indices.

### Outcomes

In addition to the calculated dietary index, the primary outcome of interest was fasting insulin (NHANES variable LBXIN) (μU/ml) as a biomarker for metabolic dysfunction. Insulin was measured on a fasting subsample of participants 12 and older who were examined in the morning session. NHANES reports using the AIA-PACK IRI, a two-site immunoenzymometric assay, performed on Tosoh AIA System analyzer.

To explore the relationship between plant-based diets and fasting insulin, we selected variables associated with insulin resistance, metabolic syndrome, and non-alcoholic fatty liver disease (NAFLD). Clinical characteristics of interest were alanine amino transferase (ALT), a possible indicator of fatty liver disease, and BMI. Waist circumference, high density lipoprotein (HDL), triglycerides, systolic and diastolic blood pressure, fasting glucose, and energy intake were additional reported characteristics. Blood pressure measurements were obtained by NHANES using a mercury sphygmomanometer and a stethoscope with an auscultatory protocol. We used the first systolic and diastolic blood pressure readings recorded.

### Statistical analyses

Statistical analyses were performed using SAS version 9.4 (SAS Institute). Fasting insulin measurements were right-skewed and were transformed to the natural log scale, hereafter called the log of fasting insulin. Continuous variables were reported as medians and interquartile ranges (25^th^ and 75^th^ percentiles). Among the quintiles of diet consumption, Cochran-Armitage, or Cochran-Mantel–Haenszel tests were used to test for trending differences in the proportions of categorical variables, sex and race. Jonckheere-Terpstra tests were used to detect trending differences among the quintiles for the continuous variables: age, BMI, waist circumference, HDL, triglycerides, fasting glucose, systolic blood pressure, diastolic blood pressure, ALT, and fasting insulin. These results are shown in Tables 3 and 4 (see pages 30 and 31). A *p* value < 0.05 was considered statistically significant.

**Table 3:** Clinical and demographic characteristics of subjects with 2-day dietary recall used to calculate PDI

			Total (*n* = 1714)				
	Overall	Q1	Q2	Q3	Q4	Q5	*P*-trend^*^
	(*n* = 1714)^a^	(*n* = 361)^b^	(*n* = 308)^c^	(*n* = 345)^d^	(*n* = 369)^e^	(*n* = 331)^f^	
*Demographics*							
Age, years	52 [36–64]	53 [35–63]	49 [33–63]	53 [36–65]	53 [38–65]	52 [38–63]	0.09
Female, %	52.23	51.8	54.55	54.2	50.68	50.76	0.51
Race/ethnicity, %							< 0.0001
Non-Hispanic White	35.59	33.24	37.99	38.55	37.67	30.51	
Non-Hispanic Black	23.75	34.9	32.79	22.32	15.18	14.2	
Mexican American	13.48	8.03	11.04	12.17	18.16	17.82	
Non-Hispanic Asian	12.08	6.37	7.47	10.43	14.63	21.45	
Other Hispanic	9.16	8.59	6.82	10.43	7.86	12.08	
Other Race—Including Multi-Racial	5.95	8.86	3.9	6.09	6.5	3.93	
*Clinical characteristics*							
BMI (kg/m**2)	28.5 [24.7–33.5]	29.5 [24.7–34.6]	29.9 [25.1–34.8]	28.2 [24.5–33.3]	28.2 [24.8–32.8]	27.5 [ 24.1–31.7]	0.0001
Waist							
Female, cm	97.5 [86.6–110.9]	101.8[88.2–114]	103[90.2–115.9]	94.9[86.2–107.2]	97.3[85.7–109]	93.5[85.5–102.4]	< .0001
Male, cm	100.1 [90.2–111.6]	101.1[91–115]	100.4[89.4–110.5]	101.4[87.9–115.1]	98.9[91.1–106.8]	100.2[90.3–110.4]	0.3
HDL, cholesterol							
Female (mg/dl)	55 [47–66]	53[46–64]	54[47–65]	56[48–67]	56[47–66]	59[50–69.5]	0.0002
Male (mg/dl)	46 [40–56]	44 [39–57]	48[40–57]	46[40–55]	46[40–54]	47[41–57]	0.62
Triglycerides (mg/dl)	94 [63–136]	94[65–132]	93[63–140]	94[61–129]	91[61–140]	97[62–136]	0.8
Fasting glucose(mg/dl)	104 [96–114]	104[96–114]	102[96–111.5]	104[97–114]	104[97–117]	104[96–114]	0.39
Systolic blood pressure (mmHg)	124 [112–136]	124[114–136]	124[114–136]	120[110–134]	124[114–136]	122[110–136]	0.22
Diastolic blood Pressure (mmHg)	72 [66–80]	74[64–80]	74[67–80]	70[64–80]	74[66–80]	72[66–80]	0.67
ALT (IU/L)	18 [13–26]	17 [12–25]	17 [12–25]	18 [13–27]	18 [13–27]	18 [14–25]	0.07
Energy intake (kcal/d)	1922 [1448–2502]	1640 [1251–2083]	1826 [1399–2412]	1899 [1470–2455]	2083 [1595–2724]	2118 [1708–2729]	< 0.0001
*Outcome variables*							
Insulin (μU/ml)	9.74 [6.20–15.56]	10.0 [6.5–15.9]	10.8[6.6–17]	9.5[6–15.3]	9.6 [6.2–14.2]	8.8 [5.9–15]	0.03
PDI	7 [3–11]	0 [(-1)-2]	4 [3–5]	7 [6–8]	10 [9–11]	15 [14–18]	

Median and interquartile range [IQR 25th-75th]

Waist circumference: obesity: men >  = 102 cm, women >  = 88 cm

*BMI* Body mass index, *PDI* Plant-based diet index

^*^ *P*-values for trend were calculated with the use of Jonckheere-Terpstra, Cochran-Armitage, or Cochran-Mantel–Haenszel tests, where appropriate

^a^BMI *n* = 1698, systolic and diastolic blood pressure *n* = 1537, ALT *n* = 1710

^b^BMI *n* = 358, Waist *n* = 351, systolic and diastolic blood pressure *n* = 310, ALT *n* = 357

^c^BMI *n* = 305, Waist *n* = 300, systolic and diastolic blood pressure *n* = 272

^d^BMI *n* = 342, Waist *n* = 338, systolic and diastolic blood pressure *n* = 314

^e^BMI *n* = 366, Waist *n* = 361, systolic/diastolic blood pressure *n* = 338

^f^BMI *n* = 327, Waist *n* = 326, systolic and diastolic blood pressure *n* = 303

**Table 4:** Clinical and demographic characteristics of subjects with 2-day dietary recall used to calculate hPDI

			Total (*n* = 1714)			
	Q1	Q2	Q3	Q4	Q5	*P*-trend^*^
	(*n* = 358)^a^	(*n* = 305)^b^	(*n* = 366)^c^	(*n* = 341)^d^	(*n* = 344)^e^	
*Demographics*						
Age, y	49 [32–63]	51 [36–63]	52 [36–63]	54 [39–65]	56 [42–65]	< 0.0001
Female, %	40	50	54	54	63	< 0.0001
Race/ethnicity, %						< 0.0001
Non-Hispanic White	38.27	38.36	34.97	38.71	27.91	
Non-Hispanic Black	37.71	28.85	22.13	19.06	11.05	
Mexican American	10.06	11.48	15.03	14.37	16.28	
Non-Hispanic Asian	0.28	6.23	11.48	14.08	28.2	
Other Hispanic	6.15	8.52	11.75	8.21	11.05	
Other Race—Including Multi-Racial	7.54	6.56	4.64	5.57	5.52	
*Clinical characteristics*						
BMI (kg/m**2)	29.3[24.9–34.6]	29.2 [25.1–34.3]	29.1 [24.8–34.2]	28 [24.6–32.7]	27.3 [23.9–31.6]	< 0.0001
Waist						
Female, cm	103.3[90.6–115.3]	101.7[90.2–113.2]	99[88.5–112.5]	95.4[86.5–106.5]	93.3[82.7–104]	< 0.0001
Male, cm	100[90.1–112.8]	100.4[89.9–112.7]	100[90.2–110.6]	102.6[91.7–111.6]	97.4[88.8–107.1]	0.31
HDL, cholesterol						
Female (mg/dl)	51[46–59]	53[46–65]	54[47–66]	57[48–67.5]	59[50–69]	< 0.0001
Male (mg/dl)	44[38–57]	46[39–55.5]	47[41–55]	47[39–56]	48[41–59]	0.07
Triglycerides (mg/dl)	93.5[64–134]	96[66–137]	92[65–140]	95[60–144]	92[59–128.5]	0.23
Fasting glucose(mg/dl)	103[95–112]	104[97–113]	104[97–115]	105[97–114]	103[96–118]	0.13
Systolic blood pressure (mmHg)	121[114–136]	124[114–136]	124[112–134]	124[112–136]	124[110–136]	0.35
Diastolic blood Pressure (mmHg)	74[66–80]	74[66–80]	72[66–80]	72[66–78]	72[66–78]	0.08
ALT (IU/L)	18 [12–27]	18 [13–25]	18 [13–26]	18 [13–27]	18 [13–25]	0.32
Energy intake (kcal/d)	2170 [1633–2707]	1984 [1491–2555]	1902 [1448–2542]	1828 [1390–2409]	1767 [1312–2276]	< 0.0001
*Outcome variables*						
Insulin (μU/ml)	10[6.7–16.5]	10.5 [6.8–16.7]	10.1 [6.4–16.3]	9.4 [6.1–15.1]	8.4 [5.6–13.7]	0.0002
hPDI	-10	-3	2	9	17	

Median and interquartile range [IQR 25th-75th]

Waist circumference: obesity: men >  = 102 cm, women >  = 88 cm

^*^ *P*-values for trend were calculated with the use of Jonckheere-Terpstra, Cochran-Armitage, or Cochran-Mantel–Haenszel tests, where appropriate

^a^BMI *n* = 352, waist *n* = 344, systolic and diastolic blood pressure *n* = 316, ALT *n* = 356

^b^BMI *n* = 302, waist *n* = 300, systolic and diastolic blood pressure *n* = 273, ALT *n* = 304

^c^BMI *n* = 362, waist *n* = 359, systolic and diastolic blood pressure *n* = 320, ALT *n* = 365

^d^BMI *n* = 338, waist *n* = 334, systolic and diastolic blood pressure *n* = 314

^e^waist *n* = 339, systolic and diastolic blood pressure *n* = 319

Associations between the log of fasting insulin and race and gender were analyzed using ANOVA. Simple log-linear regression was used to assess associations between index scores and the primary outcome variable, the log of fasting insulin. The initial multiple log-linear regression analysis considered PDI, age, race, gender, BMI, and ALT as independent predictors of the log of fasting insulin. Based on simple log-linear regression, age was non-significant (*p* = 0.99) and removed from further consideration. In the resulting multivariable log-linear model including PDI, race, gender, BMI, and ALT as predictors, gender (*p* = 0.24) and race (*p* = 0.25) were non-significant and subsequently were removed. Our final model included only PDI, BMI, and ALT as predictors of the log of insulin. To report the effects of changes of one unit in each predictor on insulin (unlogged), we took the antilog of the absolute value of each estimated regression coefficient and multiplied by 100%, thereby estimating the expected percent change (either increase or decrease) in insulin per unit change in a predictor.

Identifying a model including hPDI as a predictor followed a similar process. The initial hPDI multiple log-linear regression analysis considered hPDI, age, race, gender, BMI, and ALT as independent predictors of the log of insulin. Based on simple log-linear regression, age was non-significant (*p* = 0.99) and was removed. In the resulting multivariable log-linear analysis with hPDI, race, gender, BMI, and ALT included as predictors, gender (*p* = 0.13) and race (*p* = 0.27) were non-significant and were removed. Our final model included only hPDI, BMI, and ALT as the predictors of the log of fasting insulin.

## Results

The distributions of demographic and clinical characteristics according to the PDI and hPDI are represented in Tables 3 and 4 (pages 30 and 31), respectively.

Analyses were based on 1,714 participants, consisting of 897(52%) women and 817 (48%) men with a median age of 52 years (IQR:36,64). In this sample, 610 (36%) were white, 407 (24%) were black, 231 (13%) were Mexican, 207 (12%) were Asian, 157 (9%) were other Hispanic, and 102 (6%) were other or mixed race.

The study population’s median BMI was 28.5 (IQR: 24.7, 33.5). Males had a median waist circumference of 100.1 cm (IQR: 90.2, 111.6), females 97.5 cm (IQR: 86.6, 110.9). Male subjects presented with a median HDL of 46 mg/dL (IQR: 40, 56), females 55 mg/dL (IQR: 47, 66). Participants had a median triglyceride of 94 mg/dL (IQR: 63, 136), fasting glucose of 104 mg/dL (IQR: 96, 114), systolic blood pressure of 124 mmHg (IQR: 112, 136), diastolic blood pressure of 72 mm Hg (IQR: 66, 80), and a median ALT of 18 IU/L (IQR: 13, 26).

Participants with higher index scores in the PDI and hPDI were more likely to have a lower BMI (*p* trend < 0.0001 and *p* trend < 0.0001, respectively). Females with the highest PDI and hPDI scores were more likely to have a lower waist circumference (*p* trend < 0.0001 and *p* trend < 0.0001) and higher HDL (*p* trend = 0.0002 and *p* trend < 0.0001, respectively) than those with lower scores. Participants with higher hPDI scores were more likely to be older (*p* trend < 0.0001) and female (*p* trend < 0.0001).

In the total sample, the PDI ranged from -7 to 28 (median: 7; IQR: -11,3). The hPDI ranged from -22 to 36 (median 3; IQR: -4, 11).

The outcome variable fasting insulin, ranging from 0.71 to 105.73 μU/mL, had a median of 9.74 μU/mL (IQR: 6.2, 15.56). Fasting insulin levels were linearly associated with the quintiles of PDI (p trend = 0.03) and hPDI (p trend = 0.0002).

Fasting insulin differed significantly between Mexican Americans versus white (15.1 μU/mL versus 11.9 μU/mL, *p* < 0.0001), Mexican Americans versus black (15.1 μU/mL versus 13 μU/mL, *p* = 0.01), and Mexican versus Asian races (15.1 μU/mL versus 11.04 μU/mL, *p* < 0.0001). Black versus Asian groups (13 μU/mL versus 11.04 μU/mL) significantly differed as well (*p* = 0.03).

### PDI

Based on a simple log-linear regression, as PDI increased, log of fasting insulin decreased (-0.0068 μU/ml ± 0.0030, CI: -0.00097, -0.013) (*p* = 0.02). Thus, a one-point increase in PDI was associated with a 0.68% decrease in insulin.

In our final log-linear multivariable model, adjusting for BMI and ALT (R^2^ = 0.35), PDI was not significantly predictive of fasting insulin (0.00019 ± 0.0024, CI: -0.0045, 0.0049) (*p* = 0.94). (Table 5) (page 31). The estimated parameter of the regression of log of fasting insulin on PDI had a relatively large standard error (0.0024) and a sign that was positive rather than negative as expected. Thus, this association based on simple regression was not robust due to multicollinearity with BMI.

**Table 5:** PDI- Multivariable analysis versus log of fasting insulin^a^

	Parameter estimate	SE	*p* value	Percent change
PDI	0.00019	0.0024	0.94	0.019
BMI	0.053	0.0019	< 0.0001	5.5
ALT (IU/L)	0.0082	0.00088	< 0.0001	0.82

^a^Tables 5 and 6 show analyses using the natural log of fasting insulin

Categorization of PDI and/or BMI did not significantly improve results.

A model adjusting for metabolic variables (age, gender, BMI, HDL, triglycerides, fasting glucose, systolic blood pressure, diastolic blood pressure, and ALT) produced similar results to the simpler model including diet indices, ALT, and BMI, which we described.

### hPDI

Based on simple log-linear regression, hPDI was inversely and significantly associated with log of fasting insulin (-0.0067 ± 0.0016, CI: -0.0099, -0.0035) (*p* < 0.0001). Thus, a one-point increase in hPDI was associated with a 0.67% decrease in insulin.

In the final multivariable model containing hPDI, BMI, and ALT (R^2^ = 0.36), as hPDI was inversely and significantly associated with the log of fasting insulin (-0.0027 μU/ml ± 0.0013, CI: -0.000087, -0.0053) (*p* = 0.04) (Table 6) (page 31). Thus, a one-point increase in hPDI was associated with a 0.27% decrease in insulin, after adjusting for BMI and ALT. Unlike the PDI analysis, after adjusting for BMI and ALT, the association of hPDI with the log of fasting insulin regression was robust. Furthermore, the estimated regression coefficients and standard errors for BMI and ALT were essentially the same as those for the prediction equation including PDI.

**Table 6:** hPDI- Multivariable analysis versus log of fasting insulin^a^

	Parameter estimate	SE	*p* value	Percent change
hPDI	-0.0027	0.0013	0.04	-0.27
BMI	0.053	0.0019	< 0.0001	5.4
ALT (IU/L)	0.0082	0.00088	< 0.0001	0.82

^a^Tables 5 and 6 show analyses using the natural log of fasting insulin

A model adjusting for metabolic variables including age, gender, BMI, HDL, triglycerides, fasting glucose, systolic blood pressure, diastolic blood pressure, and ALT produced similar results.

## Discussion

Single nutrient analysis of diet does not realistically represent the way people eat, even though intake of a single nutrient is sometimes associated with a certain dietary pattern, e.g., low fat intake may be associated with increased plant intake. Foods are consumed in combinations so analyzing the dietary pattern is a more realistic approach to capturing eating behavior [33]. The interviewer-administered 24-h dietary recall is one method of capturing dietary patterns. It is less biased than the less costly FFQ and adjusts for random error due to day-to-day variation by repeat administrations, making it a more precise measure of dietary recall [34, 35]. While the FFQ has been used in many large studies e.g., Women’s Health Initiative, Jackson Heart study, and National Health and Nutrition Examination Study prior to 2003 to examine dietary patterns, it has also been shown to poorly capture the nutrient intake of some racial minorities and age groups [36, 37]. Our findings indicate that repeated measures 24-h dietary recall data can be used to create dietary indices, and our results are consistent with similar previous studies studying plant-based diet indices.

In this cross-sectional study, we found that greater adherence to a plant-based diet (as measured by PDI and hPDI) was associated with a lower fasting insulin. This relationship was most significant when healthy plant-based foods were consumed. Only the healthy plant-based diet index had an association with fasting insulin which was robust when BMI and ALT were included in the model. The healthfulness of the plant-based diet is essential in producing its positive health outcomes.

Multiple mechanisms may contribute to the benefits of a plant-based diet. In addition to being lower in energy density, plant-based diets provide increased fiber, unsaturated fatty acids, and antioxidants, while being low in saturated fat; animal products are higher in saturated fatty acids. The buildup of saturated fatty acid intermediates in muscle cells interferes with cell signaling, contributing to insulin resistance [8, 38, 39]. Vegan diets have low saturated fatty acids and high soluble and insoluble fiber content and are associated with reductions in insulin resistance [11].

Water soluble fiber forms a viscous gel in the intestinal lumen, which decreases the absorption of carbohydrates, fats, and cholesterol. Insoluble fiber is not fully digested and aids in generating fecal mass, decreasing constipation. Together soluble and insoluble fiber in plant foods is responsible for increasing satiety, while delaying gastric emptying and increasing nutrient absorption. This higher fiber content is believed to be a mechanism for weight loss, reduction of visceral and subfascial fat in muscle, and consequently, improved glucose homeostasis, as seen among subjects consuming a plant-based diet [21, 40].

We found that participants with the highest PDI and hPDI scores, had the lowest BMI (*p* trend < 0.0001). A vegetarian diet can reduce body weight, BMI, and waist circumference. Vegan diets produce the greatest weight loss compared to omnivorous diets [41–44]. A meta-analysis of four intention-to-treat studies found a 3.4 kg weight loss among vegetarian participants [43]. Likewise, a randomized controlled trial assigned participants to one of five diets (omnivorous, semi-vegetarian, pescatarian, vegetarian, vegan) for 2 months following diet training and found the greatest weight loss among vegans, followed by vegetarians, pescatarians, semi-vegetarians, and lastly omnivores. At both 2 and 6 months, the weight loss was significantly different in the vegan group versus the omnivorous group [45]. Well-planned vegan and vegetarian diets can be used for weight loss and for the prevention of disease [1, 8, 9].

Unlike studies by Satija et al., the study by Kim et al. of all-cause mortality reported that a minimum threshold of healthy food had to be consumed before health benefits were measurable [4, 5, 10]. This previous result is consistent with our finding that hPDI and not PDI was significantly negatively associated with fasting insulin in a multivariable analysis. Satija et al. used health professional data; Kim et al. used NHANES data. The health professional participants may have eaten a diet consisting of higher-quality plant foods (whole grains, fruits, vegetables, nuts, legumes, vegetable oils, tea and coffee) than the NHANES general population sample. Consuming healthy plant-based food, not just food free of animal products, is important to achieving health benefits [34].

Participants in quintile 5 of the PDI demonstrated an increase in triglycerides. Dietary fiber is inversely related to triglyceride levels. However, in some subjects, vegan or vegetarian refined carbs can result in increased triglycerides due to the excess insulin release. Whole “healthy” plant foods minimized this triglyceride response. With both the PDI and hPDI, the association between fasting insulin and triglycerides was not statistically significant. Previous studies have found an inverse association between healthy plant-based diets and dyslipidemias [46]. This was not observed in this limited sample.

### Limitations

This study did not consider physical activity, sleep, or other lifestyle behaviors which contribute to weight regulation. Regular exercise is known to decrease insulin resistance and improve insulin sensitivity. Likewise, alcohol consumption and smoking were not considered. Although these factors will alter insulin sensitivity, they are beyond the scope of this study. The omission of lifestyle factors including sleep, physical activity, smoking, education, socioeconomic status, and family history of diabetes may bias our results. However, we believe that the results of our exploratory study contribute to the body of evidence supporting healthy diet and demonstrate that these indices can be developed using dietary recall data. Furthermore, future studies utilizing these indices should include many confounding variables in the models to better account for bias.

Food recall data is imprecise, but having multiple days of recall and interviews reported by trained staff improves precision. Additionally, the duration for which participants followed the diet reported is unknown. Because only a two-day snapshot of consumption and one fasting insulin measurement per participant were observed, this study is limited by its cross-sectional design and by possible measurement errors associated with the collection of dietary data and laboratory measurement.

We used two 24-h NHANES dietary recalls gathered on rotating days of the week to produce estimates of dietary intake. In future studies we recommend the use of the NCI (National Cancer Institute) statistical modeling to better estimate usual intake distributions and address bias created by individuals eating certain foods only occasionally [30].

Sample characteristics may limit generalizability. Racial and ethnic groups have been oversampled across different NHANES cycles to produce more reliable population subgroup estimates. The oversampled subgroups in the 2017–2018 survey cycle were Hispanic persons, non-Hispanic black persons, Non-Hispanic Asian persons, non-Hispanic white and other persons at or below 185 percent of the Department of Health and Human Services (HHS) poverty guidelines, and non-Hispanic white and other (people reporting races other than black, Asian, or white) participants aged 80 years and older. Survey weights were not used in this study.

Dairy could not be included as NHANES did not separate animal from non-mammal dairy products. We believe this could have significantly impacted our overall findings. Dairy intake’s association with insulin, lipids, and inflammatory biomarkers has been varied. Additionally, the role of low-fat versus regular fat dairy in type 2 diabetes has been controversial. In a recent study, cheese but not milk or yogurt was found to be inversely associated with increased coronary artery calcium [47]. The health effects of conventional dairy versus non-mammal milk and high versus low fat products should be further studied.

## Conclusion

In this his exploratory study, we found a modest inverse association between healthy plant-based diet and fasting insulin in suggesting that perhaps the quality of the plant food is essential in conferring health benefits.

We believe that more inclusive dietary data including the segregation of dairy products by origin would be worthy of further analysis using the healthy plant-based diet index. Healthy plant-based foods should be recommended for all and especially individuals who are experiencing elevations in insulin or glucose.

## Data Availability

The datasets supporting the conclusions of this article are available in the following pages: CDC NHANES 2017–2018: https://wwwn.cdc.gov/nchs/nhanes/continuousnhanes/default.aspx?BeginYear=2017 USDA Agricultural Research Service, FPED 2017–2018: https://www.ars.usda.gov/northeast-area/beltsville-md-bhnrc/beltsville-human-nutrition-research-center/food-surveys-research-group/docs/fped-databases/

## Abbreviations

ALT: Alanine aminotransferase
BMI: Body mass index
FFQ: Food frequency questionnaire
FPED: Food Patterns Equivalents
NHANES: National Health and Examination Survey
HDL: High density lipoprotein cholesterol
hPDI: Healthful plant-based diet index
IQR: Inter-quartile range
PDI: Plant-based diet index
Q: Quintile
USDA: United States Department of Agriculture Database
